# The role of airway inflammation: effectiveness of mepolizumab in asthma with and without chronic rhinosinusitis and nasal polyps

**DOI:** 10.36416/1806-3756/e20250422

**Published:** 2026-08-13

**Authors:** Francesco Ardesi, Marco Vanetti, Rosella Centis, Martina Zappa, Patrizia Pignatti, Enrico Heffler, Stefania Gallo, Giulia Franzini, Denise Rossato Silva, Giovanni Battista Migliori, Antonio Spanevello, Dina Visca

**Affiliations:** 1. Dipartimento di Medicina e Riabilitazione Cardiorespiratoria, Istituti Clinici Scientifici Maugeri IRCCS, Tradate, Italia.; 2. Dipartimento di Medicina e Chirurgia, Università dell’Insubria, Varese, Italia.; 3. Servizio di Epidemiologia Clinica delle Malattie Respiratorie, Istituti Clinici Scientifici Maugeri IRCCS, Tradate, Italia.; 4. Servizio di Allergologia e Immunologia, Istituti Clinici Scientifici Maugeri IRCCS, Pavia, Italia.; 5. Upload (Upper and Lower Airways Inflammatory Diseases) Research Center, Università dell’Insubria, Varese, Italia.; 6. Dipartimento di Scienze Biomediche, Humanitas University, Pieve Emanuele, Milano, Italia.; 7. Centro di Medicina personalizzata, asma e allergologia, Humanitas Research Hospital, Milano, Italia.; 8. Dipartimento di Otorinolaringoiatria, Ospedale di Circolo e Fondazione Macchi, ASST Sette Laghi, Varese, Italia.; 9. Faculdade de Medicina, Universidade Federal do Rio Grande do Sul - UFRGS - Porto Alegre (RS) Brasil.

**Keywords:** Asthma, Nasal polyps, Inflammation, Antibodies, monoclonal, humanized

## Abstract

**Objective:**

Severe eosinophilic asthma (SEA) is often associated with chronic rhinosinusitis with nasal polyps (CRSwNP) characterized by type 2-driven eosinophilic inflammation. We sought to investigate the effect of mepolizumab on relevant clinical, functional, and inflammatory endpoints in naïve SEA patients with or without nasal polyps evaluated through induced sputum and followed for 12 months.

**Methods:**

We conducted an exploratory observational real-world cohort study including biologic-naïve SEA patients who initiated treatment with mepolizumab at an asthma referral center in Tradate, Italy. Clinical, functional, and inflammatory parameters were assessed at baseline and after 12 months. Induced sputum was used to evaluate airway inflammation. Patients were stratified by presence or absence of CRSwNP.

**Results:**

Forty patients were included, 27/40 (67.5%) had CRSwNP. After 12 months, significant reductions were observed in annual exacerbations (median, 2.0 vs. 0.0; adjusted p value [p-adj] < 0.001), maintenance oral corticosteroid use (5.0 vs. 0.0 mg; p-adj = 0.004), blood eosinophil count (543 vs. 55 cells/µL; p-adj < 0.001), and sputum eosinophil % (39.0% vs. 1.3%; p = 0.004). Asthma control and FEV_1_ improved significantly. In patients with CRSwNP, sinonasal symptoms markedly improved (p-adj = 0.004). No post-treatment differences in airway eosinophilia were detected between the groups. Overall, 77.5% achieved clinical remission in accordance with Severe Asthma Network Italy Delphi consensus criteria.

**Conclusions:**

Mepolizumab provided substantial clinical, functional, and anti-inflammatory benefits in SEA, regardless of CRSwNP status. Despite higher baseline eosinophilia, patients with CRSwNP showed no clear differences in outcomes when compared with those without CRSwNP, supporting the concept of united airway disease. These findings highlight the value of induced sputum as a noninvasive tool and support integrated, comorbidity-driven approaches in severe asthma management.

## INTRODUCTION

Severe asthma is a heterogeneous chronic airway disease characterized by persistent respiratory symptoms and frequent exacerbations despite high-dose inhaled corticosteroids and additional controller therapy.^1^

Approximately 70% of patients with severe asthma have evidence of high levels of T2 inflammation. T2 biomarkers have been identified as predictors of asthma severity and worse clinical control and lung function decline.^2^

The disease burden is further amplified by comorbidities (e.g., airway-related comorbidities, gastroesophageal reflux disease, obesity, and cardiovascular diseases), which affect a substantial proportion of patients with severe asthma and contribute to worse disease control and respiratory symptoms.^3^ ^,^ ^4^

Airway-related comorbidities, such as chronic rhinosinusitis with or without nasal polyposis, allergic rhinitis, bronchiectasis, and vocal cord dysfunction, are among the most common and troublesome comorbidities in severe asthma.^3^ In Italy, 42.6% of severe asthma patients have chronic rhinosinusitis with nasal polyps (CRSwNP) leading to higher rates of asthma exacerbations, increased systemic corticosteroid use, and more persistent airway obstruction.^5^

Severe eosinophilic asthma (SEA) and CRSwNP share similar T2 inflammation, with IL-5 playing a central role in eosinophilic infiltration and tissue remodeling in the upper and lower airways. Airway eosinophilic inflammation contributes to airway hyperresponsiveness, mucus plugging, and chronic airway remodeling, with increased risk of exacerbations.^6^

Mepolizumab has demonstrated efficacy in patients with SEA in randomized clinical trials and real-life studies.^7^ ^-^ ^10^ Notably, patients with comorbid nasal polyps may experience even greater clinical benefit, including higher rates of super-response and clinical remission, supporting a comprehensive treatment approach for patients with comorbid eosinophilic disease.^11^ ^,^ ^12^

Beyond its clinical benefits, mepolizumab has demonstrated efficacy in promptly reducing blood eosinophilic inflammation,^8^ confirming blood eosinophilia as one of the most useful and feasible biomarkers.^2^ On the other hand, bronchial inflammation, which is considered a hallmark of SEA, has been less extensively investigated, mainly due to limited availability of noninvasive techniques and/or restricted applicability of invasive tools in routine clinical practice, limiting their use mostly to research settings. Nevertheless, induced sputum remains the gold standard to study airway inflammation noninvasively.^13^ To date, data on the magnitude of benefit in SEA patients treated with antieosinophilic biologic agents, particularly regarding bronchial inflammation, have been evaluated in a limited number of studies, whose evidence is summarized in Chart 1.^7^ ^,^ ^8^ ^,^ ^14^ ^-^ ^22^ In particular, no study has evaluated airway inflammation with the use of induced sputum in SEA, comparing patients with or without CRSwNP before and after biologic therapy. 

**Chart 1:** Evidence on airway inflammation in severe asthma patients treated with mepolizumab.

Study	Objective	Study design	Inclusion criteria	Study subjects	Comments	Information not included
Domvri et al.^14^	Impact of mepolizumab on airway remodeling	Prospective observational study	Late-onset severe eosinophilic asthma patients with fixed obstruction	39	Real-life Bronchial biopsies	No induced sputum No comparison CRSwNP vs. without CRSwNP
Gerday et al.^15^	Role of baseline sputum eosinophils in identifying super-responders	Prospective observational study	Adult severe eosinophilic asthma patients	106	Real-life Mepolizumab or benralizumab Induced sputum	No comparison CRSwNP vs. without CRSwNP
Moermans et al.^16^	Role of mediators of the type 2 cascade in the sputum as predictors of remission	Retrospective observational study	Adult severe asthma patients	52	Real-life Induced sputum Mepolizumab or reslizumab	No comparison CRSwNP vs. without CRSwNP
Šokić et al.^17^	Blood and induced sputum eosinophil evaluation in responders and nonresponders on anti-IL-5	Retrospective observational study	Adult severe asthma patients	17	Real-life Induced sputum only at follow-up Mepolizumab or benralizumab	No induced sputum at baseline No comparison CRSwNP vs. without CRSwNP
Crimi et al.^18^	Effectiveness of mepolizumab in severe eosinophilic asthma with and without bronchiectasis	Retrospective observational study	Adult severe eosinophilic asthma patients	32	Real-life Induced sputum Bronchiectasis vs no bronchiectasis	No comparison CRSwNP vs. without CRSwNP
Detoraki et al.^19^	Efficacy of mepolizumab in severe eosinophilic asthma with CRSwNP	Prospective observational study	Adult severe eosinophilic asthma patients with CRSwNP	44	Real-life Evaluation of nasal cytology	No induced sputum No comparison CRSwNP vs. without CRSwNP
McDowell et al.^20^	Evaluation of asthma exacerbations on mepolizumab	Prospective observational study	Adult severe asthma patients	140	Real-life Induced sputum	No comparison CRSwNP vs. without CRSwNP
Mukherjee et al.^21^	Observe response to anti-IL-5 and evaluate predictors of suboptimal response	Prospective observational study	Adult moderate-to-severe asthma patients	250	Real-life Induced sputum Mepolizumab or reslizumab	No comparison CRSwNP vs. without CRSwNP
Schleich et al.^22^	Efficacy of mepolizumab in severe eosinophilic asthma	Prospective observational study	Adult severe eosinophilic asthma patients	116	Real-life Induced sputum	No comparison CRSwNP vs. without CRSwNP
Pavord et al.^7^	Efficacy and safety of mepolizumab	Multicenter, double-blind, placebo-controlled trial	Severe eosinophilic asthma patients (12-74 years of age)	621	Different doses of mepolizumab Induced sputum	No real-life No comparison CRSwNP vs. without CRSwNP
Haldar et al.^8^	Effect of mepolizumab on frequency of exacerbations	Randomized, double-blind, placebo-controlled, parallel-group study	Patients with refractory asthma and evidence of eosinophilic airway inflammation	61	Induced sputum	No real-life No comparison CRSwNP vs. without CRSwNP

CRSwNP: chronic rhinosinusitis with nasal polyps.

The present study was aimed at evaluating the effect of mepolizumab on relevant clinical, functional, and inflammatory endpoints in naïve SEA patients with and without nasal polyps investigated through induced sputum and followed for 12 months, focusing on airway inflammation. 

## METHODS

### Study design and setting

We conducted an exploratory observational real-world cohort study including severe asthma patients who initiated treatment with mepolizumab between May 2019 and June 2024 at the *Istituti Clinici Scientifici Maugeri IRCCS*, an Italian national referral center specialized in severe asthma management located in Tradate, Italy. Demographic, clinical, and biological data were collected at baseline and after 1 year of treatment and were entered into a dedicated deidentified electronic database. 

The study was performed in accordance with the Declaration of Helsinki and was approved by the Institutional Review Board of the *Istituti Clinici Scientifici Maugeri IRCCS* , Tradate (identifier: p3/16). 

### Patients and study procedures

We evaluated a cohort of outpatients diagnosed with SEA according to the GINA guidelines.^1^ We enrolled all consecutive biologic-naïve patients who initiated treatment with mepolizumab in accordance with Italian Medicines Agency eligibility criteria and who completed a 1-year follow-up period. Collected variables included baseline demographics (age, sex, BMI, and smoking history), age at asthma onset (childhood-onset asthma: < 18 years of age; adult-onset asthma: ≥ 18 years of age), atopic status, symptom control, frequency of acute exacerbations, pharmacological treatment, and comorbidities. Data were collected at baseline (T0) and after 12 months of treatment (T12).

Atopy was defined in accordance with World Allergy Organization criteria and assessed in routine clinical practice via skin prick testing for common inhalant allergens and/or measurement of total and specific serum IgE. The diagnosis of CRSwNP was confirmed by otorhinolaryngologists. 

Asthma control was routinely evaluated with the six-item Asthma Control Questionnaire (ACQ-6) and the Asthma Control Test. Exacerbations were recorded and defined as episodes of worsening symptoms and lung function relative to the patient’s usual baseline, requiring oral corticosteroid therapy. 

Pulmonary function tests were performed with a Pony FX spirometer (COSMED, Chicago, IL, USA), including FEV_1_ (in L and % predicted), FVC (in L and % predicted), the FEV_1_/FVC ratio, and FEF_25-75%_, in accordance with American Thoracic Society/European Respiratory Society standards. All values were standardized by using the Global Lung Function Initiative reference equations. Fractional exhaled nitric oxide (FeNO) was measured with an FeNO monitoring system (Vivatmo *pro*; Bosch Healthcare Solutions GmbH, Waiblingen, Germany) in accordance with European Respiratory Society/American Thoracic Society standards.^23^

Data on central airway inflammation were noninvasively obtained from induced sputum analyses performed in our routine clinical practice in patients without exacerbations in the preceding month. Sputum induction was carried out using 4.5% hypertonic saline inhalation and processed with dithiothreitol, in accordance with European Respiratory Society guidelines.^13^ Response to treatment after 12 months was evaluated according the criteria defined by a Delphi consensus from the Severe Asthma Network Italy (SANI).^24^

### Statistical analysis

Categorical variables were expressed as absolute numbers and percentages, whereas continuous variables were summarized as medians with interquartile ranges. Data normality distribution was assessed by the Shapiro-Wilk test and graphical representation (quantile-quantile plots). The Mann-Whitney U test and the chi-square test (with Yates’ continuity correction) were used to compare SEA with and without CRSwNP. Wilcoxon and McNemar tests were used to compare groups at different time points. The Benjamini-Hochberg method was applied to adjust significance values from inter- and intragroup T0-T12 comparisons. It was not used for baseline variables because they were not considered study endpoints. Statistical threshold was set at 0.05. All analyses were performed with the IBM SPSS Statistics software package, version 26 (IBM Corporation, Armonk, NY, USA) and R software, version 4.2.3 (The R Project for Statistical Computing, Vienna, Austria). 

## RESULTS

A total of 40 SEA patients receiving mepolizumab were included in the analysis; among them, 27 (67.5%) had concomitant CRSwNP and 13 (32.5%) did not (Table 1). Based on the presence of CRSwNP, patients were stratified into two groups and considered separately in subsequent analyses. 

**Table 1:** Baseline characteristics of severe eosinophilic asthma with and without chronic rhinosinusitis with nasal polyps.^a^

Variable		With CRSwNP (n = 27)	Without CRSwNP (n = 13)	p value	Total sample (N = 40)
Age at treatment initiation, years		58.0 [48.0-62.0]	57.0 [50.5-64.0]	0.840	58.0 [48.8-62.0]
Male		12 (44.4)	3 (23.08)	0.298	15 (37.5)
BMI, kg/m^2^		24 [22.3-27.3]	30.8 [21.9-35.8]	0.103	24.9 [22.2-29.8]
Smoking status	Nonsmoker	14 (51.9)	9 (69.2)	0.484	23 (57.5)
	Former smoker	13 (48.2)	4 (30.8)		17 (42.5)
Age at respiratory symptom onset, years		41.0 [20.0-50.0]	46.0 [21.5-54.5]	0.696	43.0 [20.3-51.5]
Adult-onset asthma		21 (77.78)	10 (76.92)	1.000	31 (77.5)
Beclomethasone HFA equivalent, mcg		600.0 [480.0-800.0]	600.0 [480.0-800.0]	0.963	600 [480.0-800.0]
IgE, kU/L		208.0 [133.0-609.0]	201.0 [53.9-633.8]	0.715	208 [91.3-609.0]
Atopy		16 (59.3)	10 (76.9)	0.316	26 (65.0)
GERD		8 (29.6)	7 (53.9)	0.175	15 (37.5)
Obesity		2 (7.4)	7 (53.9)	0.002	9 (22.5)
Arterial hypertension		8 (29.6)	6 (46.2)	0.480	14 (35.0)
Bronchiectasis		7 (25.9)	5 (38.5)	0.476	12 (30.0)
Emphysema		3 (11.1)	2 (13.4)	1.000	5 (12.5)
OSA		4 (14.8)	7 (53.9)	0.020	11 (27.5)

SEA: severe eosinophilic asthma; CRSwNP: chronic rhinosinusitis with nasal polyps; HFA: extrafine hydrofluoroalkane; GERD: gastroesophageal reflux disease; and OSA: obstructive sleep apnea. ^a^Data presented as n (%) or median [IQR].

The overall median age at treatment initiation was 58 years [IQR, 48-62], with 37.5% being male; no significant differences were found between the two groups in terms of age or sex distribution (p = 0.840 and p = 0.298, respectively). Median BMI was 24.9 kg/m^2^ [IQR, 22.2-29.8], with a nonsignificant trend toward higher values in patients without CRSwNP (30.8 [21.9-35.8] vs. 24.0 [22.3-27.3]; p = 0.103). Baseline treatment with high-dose inhaled corticosteroids was similar across groups. Regarding comorbidities, gastroesophageal reflux disease was numerically more frequent in the non-CRSwNP group, although not statistically significant. In contrast, obesity (53.9% vs. 7.4%; p = 0.002) and obstructive sleep apnea (53.9% vs. 14.8%; p = 0.020) were significantly more prevalent among patients without CRSwNP. No significant differences emerged for arterial hypertension, bronchiectasis, or emphysema (Table 1). 

**Figure 1:**
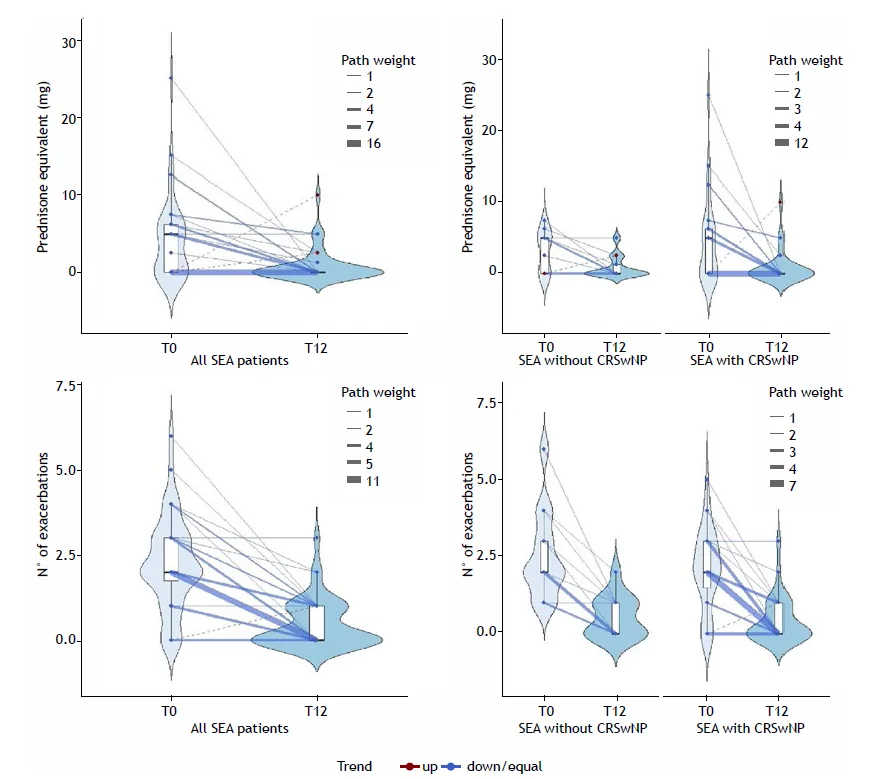
Reduction in the median number of exacerbations and maintenance oral corticosteroid dose from baseline (T0) and after 12 months of treatment with mepolizumab (T12). SEA: severe eosinophilic asthma; and CRSwNP: chronic rhinosinusitis with nasal polyps.

At T12, significant clinical benefits were observed in the overall population (Table 2). The annual number of exacerbations decreased markedly from a median of 2.0 [1.3-3.0] to 0.0 [0.0-1.0; adjusted p value (p-adj) < 0.001]. Consistently, the median prednisone-equivalent dose decreased from 5.0 mg [0.0-6.3] to 0.0 mg [0.0-0.0; p-adj = 0.004; Figure 1]. Asthma control significantly improved: median ACQ-6 scores decreased from 0.8 [0.2-2.0] to 0.2 [0.0-0.5; p-adj = 0.006], and Asthma Control Test scores increased from 22.5 [18.0-24.0] to 25.0 [22.3-25.0; p-adj = 0.005]. In patients with CRSwNP, sinonasal symptoms improved significantly, with 22-item Sino-Nasal Outcome Test (SNOT-22) scores decreasing from 44.0 [18.0-72.5] to 16.0 [4.8-34.0; p-adj = 0.004]. Blood eosinophils were significantly reduced in the entire cohort (543 [350-785] vs. 55 [38-91]; p-adj < 0.001), with consistent findings in both subgroups. Induced sputum analysis showed a marked decline in eosinophil % (39.0 [3.0-68.9] vs. 1.3 [0.7-14.8]; p-adj = 0.004). After 12 months of treatment, 35% (14/40) of the patients were not able to expectorate. No significant changes were observed in blood neutrophil counts or FeNO levels. Lung function tests revealed a significant improvement in FEV_1_% (78.5 [66.8-92.1] vs. 89.0 [74.2-103.2]; p-adj = 0.020), FEV_1_/FVC% (69.5 [63.2-77.3] vs. 73.4 [66.9-78.3]; p-adj = 0.024), and FEF_25-75%_ (58.1 [42.5-92.6] vs. 71.0 [46.1-104.0]; p-adj = 0.032). 

**Table 2:** Comparison of clinical, functional, and inflammatory characteristics between baseline and after 12 months of treatment with mepolizumab.^a^

	Total sample (N = 40)				With CRSwNP [n = 27]			Without CRSwNP (n = 13)		
Variable	T0	T12	p-adj	T0	T12	p-adj	T0	T12	p-adj
No. of exacerbations in the previous year	2.0 [1.3-3.0]	0.0 [0.0-1.00]	0.000	2.0 [1.0-3.0]	0.0 [0.0-1.0]	0.001	2.0 [1.5-3.5]	0.0 [0.0-1.0]	0.009
mOCS, prednisone equivalent mg	5.0 [0.0-6.3]	0.0 [0.0-0.0]	0.004	5.0 [0.0-6.3]	0.0 [0.0-0.0]	0.021	5.0 [0.0-5.0]	0.0 [0.0-1.9]	0.050
SNOT-22	27.5 [9.8-64.3]	15.0 [5.0-28.0]	0.003	44.00 [18.0-72.5]	16.0 [4.8-34.0]	0.004	10.0 [6.0-28.0]	10.0 [3.5-23.5]	0.355
ACQ-6	0.8 [0.2-2.0]	0.2 [0.0-0.5]	0.006	1.33 [0.2-2.3]	0.0 [0.0-0.3]	0.004	0.3 [0.0-1.5]	0.2 [0.0-1.0]	0.964
ACT	22.5 [18.0-24.0]	25 [22.3-25.0]	0.005	20.0 [18.0-24.0]	25.0 [23.0-25.0]	0.007	23.0 [19.0-24.0]	23.0 [21.5-25.0]	0.487
Blood neutrophil count, 10^9/L	3,985 [3,221-4,966]	3,814 [3,024-4,699]	0.545	3,927 [3,218-4,748]	3,723 [2,794-4,569]	0.575	4,245 [3,150-6,893]	3,937 [3,273-5,043]	0.743
Blood eosinophil count, 10^9/L	543 [350-785]	55 [38-91]	0.000	640 [422-846]	59 [39-91]	0.000	353 [284-495]	43 [36-89]	0.008
FEV_1_, % predicted	78.5 [66.8-92.1]	89.0 [74.2-103.2]	0.020	84.1 [67.0-97.9]	92.7 [80.1-106.0]	0.030	77.9 [56.2-87.3]	75.5 [64.1-96.0]	0.344
FVC, % predicted	89.7 [78.4-101.5]	93.0 [83.1-103.1]	0.250	93.9 [80.7-105.6]	97.3 [88.0-109.1]	0.303	82.7 [73.8-93.3]	89.0 [78.3-94.4]	0.487
FEV_1_/FVC, % predicted	69.5 [63.2-77.3]	73.4 [66.9-78.3]	0.024	69.0 [63.0-76.0]	75.1 [68.0-78.3]	0.030	71.0 [60.5-80.3]	67.50 [57.0-81.8]	0.449
FEF_25-75%_, % predicted	58.1 [42.5-92.6]	71 [46.1-104.0]	0.032	57.5 [41.9-99.3]	87.0 [59.1-106.2]	0.030	58.9 [42.0-84.1]	40.7 [34.6-99.9]	0.650
FeNO, ppb	55.0 [27.3-76.0]	55.0 [22.0-74.0]	0.487	68.5 [36.3-88.8]	48.50 [21.50-76.3]	0.370	37.0 [14.3-61.5]	56.00 [22.0-68.0]	0.559
Induced sputum neutrophils, %^b^	32.0 [11.6-62.3]	72.8 [31.5-94.3]	0.030	18.30 [7.2-51.3]	44.10 [26.2-86.8]	0.075	64.0 [19.9-89.2]	85.20 [71.2-95.9]	0.250
Induced sputum eosinophils, %^b^	39.0 [3.0-68.9]	1.3 [0.70-14.8]	0.004	48.40 [20.6-71.5]	2.10 [0.6-38.1]	0.010	2.6 [0.2-38.7]	1.20 [0.8-4.8]	0.271

CRSwNP: chronic rhinosinusitis with nasal polyps; p-adj: Benjamini-Hochberg adjusted p value; mOCS: maintenance oral corticosteroids; SNOT-22: 22-item Sino-Nasal Outcome Test; ACQ-6: six-item Asthma Control Questionnaire; ACT: Asthma Control Test; FeNO: fractional exhaled nitric oxide; T0: at baseline; and T12: after 12 months of treatment with mepolizumab. ^a^Data presented as median [IQR]. ^b^Induced sputum: all patients 40/40 (100%) at T0 and 26/40 (65%) at T12; with CRSwNP 17/27 (63%) and without CRSwNP 9/13 (69%) at T12.

In accordance with the SANI Delphi consensus criteria, 77.5% (31/40) of the patients reached clinical remission: 47.5% (19/40) achieved complete remission and 30.0% (12/40) achieved partial remission, whereas 22.5% (9/40) did not meet remission criteria. Two patients switched from mepolizumab to another biologic treatment because of persistent uncontrolled CRSwNP and inadequate treatment response, respectively. 

**Figure 2:**
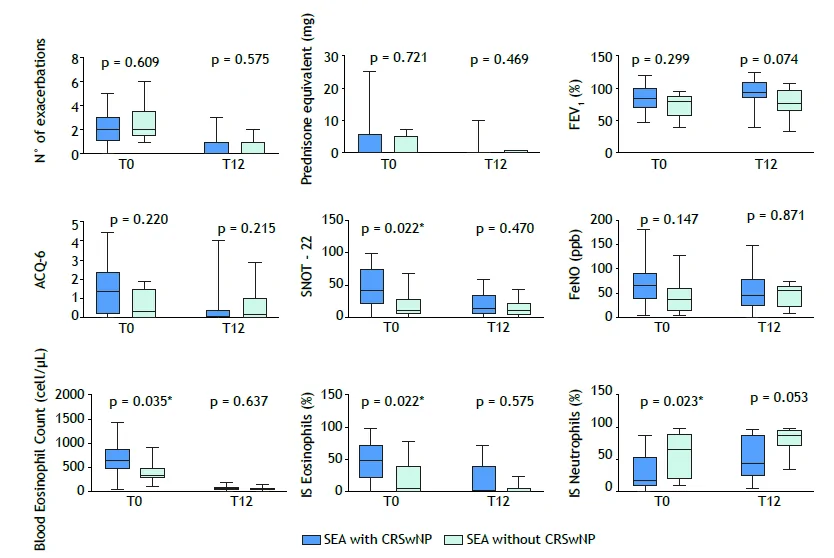
Comparison of clinical, functional, and inflammatory characteristics in severe eosinophilic asthma (SEA) with and without chronic rhinosinusitis with nasal polyps (CRSwNP) at baseline (T0) and after 12 months of treatment with mepolizumab (T12). p: Benjamini-Hochberg adjusted p value; ACQ-6; six-item Asthma Control Questionnaire; SNOT-22: 22-item Sino-Nasal Outcome Test; FeNO: fractional exhaled nitric oxide; and IS: induced sputum.

Comparing outcomes at T0 and T12 between groups (Figure 2), we found no differences in terms of number of exacerbations in the previous year, use of maintenance oral corticosteroids, FEV_1_%, ACQ-6 scores, or FeNO (p-adj > 0.05). On the other hand, patients with CRSwNP exhibited higher SNOT-22 scores, blood eosinophil count, and induced sputum eosinophils (%), as well as lower induced sputum neutrophils (%; p-adj < 0.05) at T0. These differences were no longer significant at T12. 

## DISCUSSION

Mepolizumab has demonstrated sustained efficacy over the past decade with significant improvements in asthma outcomes reported in randomized controlled trials and real-world studies.^2^ ^,^ ^12^ ^,^ ^22^ ^,^ ^25^ ^-^ ^31^ Our real-world experience provides new insights into airway inflammation through the assessment of eosinophilia not only in peripheral blood but also in induced sputum in SEA patients with and without nasal polyps. We observed that patients with concomitant CRSwNP exhibited higher sputum eosinophil levels prior to initiating treatment with mepolizumab. However, after 12 months of treatment, no differences in sputum eosinophil levels were detected between patients with and without nasal polyps, underscoring its capacity to reduce eosinophilic inflammation at the systemic and airway levels. 

Our study cohort comprised 40 severe asthma patients who were naïve from previous biologic treatment and who fulfilled Italian Medicines Agency eligibility criteria for mepolizumab treatment. Consistent with previous reports,^5^ ^,^ ^6^ we observed a high prevalence of CRSwNP (67.5%). According to published evidence, the presence of nasal polyps in severe asthma delineates an endotype characterized by persistent eosinophilic inflammation, likely sustained by synergistic type 2 cytokine activity, which promotes eosinophil recruitment and survival in upper and lower airway tissues.^6^ Based on this rationale, we sought to evaluate the impact of mepolizumab on inflammatory biomarkers and the longitudinal evolution of airway inflammation in our real-world cohort. 

Initially, we assessed the overall efficacy of mepolizumab, confirming its benefits across multiple asthma-related outcomes. After 12 months of treatment, we observed a significant improvement in asthma control questionnaire scores (p-adj = 0.006) and in lung function (FEV_1_%; p-adj = 0.020). Moreover, we observed a marked reduction in the median number of exacerbations (p-adj < 0.001) and maintenance oral corticosteroid use (p-adj = 0.004). From an inflammatory perspective, blood and sputum eosinophilia (p-adj < 0.001 and p-adj = 0.004, respectively) decreased, whereas FeNO remained unchanged, likely reflecting mepolizumab’s selective anti-IL-5 mechanism of action. Interestingly, we observed that 35% of patients were unable to expectorate after 12-month treatment. Similar results were obtained with another antieosinophilic therapy (benralizumab),^32^ likely resulting from an overall reduction in central airway inflammation. 

Although current evidence focuses on clinical outcomes such as exacerbations, lung function, and quality of life, data on the capability of spontaneous or induced sputum after initiation of biologic treatment are, to the best of our knowledge, still lacking. Further studies are warranted to determine whether induced sputum could improve clinical management during the follow-up of SEA patients on antieosinophilic biologic treatment, also in the presence of CRSwNP. 

Only one patient in the present study switched from mepolizumab to another biologic agent due to persistent uncontrolled CRSwNP, and another due to an inadequate treatment response. Moreover, 77.5% of our patients reached clinical remission in accordance with SANI Delphi consensus criteria^24^; 47.5% of the patients achieved complete clinical remission, and 30% achieved partial clinical remission. Only 22.5% of the patients did not meet those criteria. Recent studies have reported remission rates of approximately 30%^12^ ^,^ ^29^ ^,^ ^33^ ^-^ ^35^ in patients with SEA when assessed by SANI-like criteria after 12 months of mepolizumab.^33^ The discrepancy between data from previous studies and our data highlights the importance of developing a unique consensus on remission criteria and a multidisciplinary approach in dedicated severe asthma centers. 

We compared patients with and without CRSwNP to investigate differences between groups at baseline, especially regarding inflammatory features. Indeed, literature on the effect of mepolizumab on airway eosinophilic inflammation in naïve asthma patients with both conditions are lacking.^7^ ^,^ ^8^ ^,^ ^14^ ^-^ ^22^ No differences in terms of asthma control, exacerbations, maintenance oral corticosteroid use, or lung function were detected. Higher sputum neutrophilic inflammation, as well as higher prevalence of obesity, gastroesophageal reflux disease, and obstructive sleep apnea, together with reduced FVC%, were observed in the non-CRSwNP cohort. These differences may, at least in part, reflect the trend toward a higher BMI in this subgroup, although the difference in BMI did not reach statistical significance. On the other hand, patients with SEA and CRSwNP exhibited significantly higher peripheral blood eosinophil counts (p-adj = 0.035) and sputum eosinophils (p-adj = 0.22), confirming evidence from previous studies.^36^ Since eosinophilia was the main significant difference between groups at baseline, it was interesting to compare this variable after 12 months of treatment. 

At T12, this difference was no longer evident, suggesting that mepolizumab is associated with a significant reduction in airway inflammation in patients with severe eosinophilic asthma, regardless of the presence of CRSwNP. 

Aware of the limitations related to our small cohort, we decided to evaluate outcome evolution across the two groups mainly descriptively. In asthma with and without CRSwNP, mepolizumab provided substantial clinical benefits in terms of reducing exacerbations and maintenance oral corticosteroid dose, confirming data from the overall population.^2^ ^,^ ^12^ ^,^ ^22^ ^,^ ^25^ ^-^ ^31^ In patients without CRSwNP, the reduction in maintenance oral corticosteroid dose reached statistical significance (p = 0.020), although this was at the threshold of significance after the Benjamini-Hochberg correction (p-adj = 0.050). Moreover, in patients without CRSwNP, asthma questionnaire scores, FEV_1_% and induced sputum eosinophilia did not reach a statistically significant improvement. We believe that these findings may be due to the small sample of this group (13/40) rather than a true lack of efficacy in those outcomes. Nevertheless, in our population, there was an overall trend toward slightly better outcomes, mainly driven by patients with CRSwNP. This finding seems to be in line with previous studies suggesting that patients with CRSwNP may achieve higher rates of super-response or complete response, highlighting a potentially enhanced clinical and anti-inflammatory benefit.^11^ ^,^ ^12^

Together, these findings support the concept of a united airway disease concept, in which interventions targeting type 2 inflammation provide benefits across upper and lower airways, endorsing mepolizumab use in severe eosinophilic asthma regardless of comorbid upper airway disease.^37^ Further studies may be useful to confirm this evidence. 

Our findings highlight the urgent need to establish a unified consensus definition of remission in severe asthma, while also providing a rationale for extending the concept beyond traditional clinical and functional dimensions. In particular, we propose that such a definition should incorporate not only clinical and functional outcomes but also inflammatory aspects, with particular attention to a significant reduction in central airway inflammation, thus aligning with the broader concept of disease modification. Given the substantial impact of upper airway diseases on asthma trajectories, an important unmet need is the integration of comorbidity-driven perspectives, particularly from the otorhinolaryngology perspective.^38^ This approach could, for example, include assessment of changes in SNOT-22 scores and objective markers of nasal inflammation along with induced sputum evaluation to assess a “united airway disease remission” also from a multidisciplinary and inflammatory standpoint, considering the disease-modifying effect of biologic treatment. 

In our CRSwNP cohort, the improvement in clinical outcomes, as measured by changes in SNOT-22 scores, is consistent with previous studies,^39^ ^,^ ^40^ highlighting its efficacy in the upper and lower airways. Within the framework of the united airway disease model, the study of local inflammatory patterns through nasal cytology could serve as a parallel to the investigation of induced sputum analysis in asthma. Indeed, a reduction in otorhinolaryngology questionnaire scores and nasal cytology eosinophilia after mepolizumab treatment has previously been demonstrated.^19^ However, there is currently no clear consensus on the use of nasal cytology in CRSwNP, and few studies have evaluated the cytological or histological changes induced by monoclonal therapies,^41^ underscoring the need for further investigation. 

The main limitations of our study include the single-center exploratory design and a relatively small population, which reflects the number of eligible patients at the referral center and may have influenced the statistical significance of some findings. Moreover, the use of maintenance oral corticosteroids in a subset of patients may have affected some inflammatory and/or functional parameters; however, this represents a common scenario in real-life settings and underscores the value of studies reflecting everyday clinical practice. 

To the best of our knowledge, this is the first study highlighting a rationale for incorporating the study of inflammation in upper and lower respiratory diseases (i.e., induced sputum analysis and nasal cytology) and comorbidity-driven assessments in future investigations, with the goal of refining treatment strategies and advancing disease-modifying approaches in severe eosinophilic asthma. 
